# M-CHAT Mexican Version Validity and Reliability and Some Cultural Considerations

**DOI:** 10.5402/2012/408694

**Published:** 2012-07-01

**Authors:** Lilia Albores-Gallo, Ofelia Roldán-Ceballos, Gabriela Villarreal-Valdes, Blanca Xochitl Betanzos-Cruz, Claudia Santos-Sánchez, Maria Magdalena Martínez-Jaime, Isaac Lemus-Espinosa, Claudia List Hilton

**Affiliations:** ^1^Research Division, Hospital Psiquiátrico Infantil “Dr. Juan N. Navarro,” Secretaría de Salud, Mexico; ^2^Epidemiology Department, Asociación Mexicana de Niños con TDA y Trastornos Asociados AC, Mexico; ^3^Psychology School, Facultad de Psicología, Universidad Regional del Sureste, Mexico; ^4^Psychology School, Universidad Autónoma Metropolitana de México, Mexico; ^5^Washington University School of Medicine, Occupational Therapy and Psychiatry, St. Louis, MO, USA

## Abstract

The Modified Checklist for Autism in Toddlers (M-CHAT) questionnaire is a brief measure available in Spanish which needs to be validated for the Mexican population. Parents of children from (1) community with typical development (TD) and (2) psychiatric outpatient unit completed the CBCL/1.5–5 and the Mexican/MM-CHAT-version. The study sample consisted of 456 children (age M = 4.46, SD = 1.12), 74.34% TD children and 26.65% with Autism Spectrum Disorders (ASD). The MM-CHAT mean score for failed key items was higher for the ASD group compared with the TD group. Internal consistency for the Mexican/M-CHAT version was .76 for total score and .70 for the 6 critical items. Correlations between the MM-CHAT and the CBCL/1.5: PDD and Withdrawn subscales and with ADI-R dimensions: B non verbal) and A were high, and were moderate with ADI-R dimensions B1 (verbal) and C The failure rate of the MM-CHAT between the groups did not reproduce all the critical items found in other studies. Although the instrument has good psychometric properties and can be used for screening purposes in primary settings or busy specialized psychiatric clinics, these results support evidence for cultural differences in item responses, making it difficult to compare M-CHAT results internationally.

## 1. Introduction

Autism spectrum disorders (ASDs) affect 1-2% of children [1–5]. Early detection is important because it allows the introduction of early intensive treatment strategies to improve the psychosocial adjustment of these children [6–13]. The development of measurements to assess the autism spectrum disorders in the last two decades has increased. Unfortunately, the cost of using these tools for clinical and research purposes has become expensive and complicated [14]. Many of these instruments are very complex and targeted to specialized professionals with experience in autism, so their use in the primary care setting in Mexico is not feasible. Furthermore, some of them require training and take enormous time to administer and score [15, 16]. Education level and skills and attributes of parents, in addition to health and educational services, have a crucial role in recognition and diagnosis of ASD. First concerns are noticed by parents at 12–24 months of age [17, 18]. These initial observations of atypical development are followed by two types of delays to seek actions. Parental delays in seeking attention are approximately 4 months, and medical delay in assigning a diagnosis (from first reported concern to medical diagnosis) is 30.1 months [19]. In Latin-American countries most parents seek initial attention through public health services [20], but only 38% receive their diagnosis through this means [19]. Furthermore, recent studies show that many children are identified in school [21] so screening instruments need to be oriented to parents, teachers, and primary medical health providers.

The Modified CHAT (M-CHAT) (Robins et al., 2001) [5] is a simple questionnaire for parents that can be completed in 10 minutes. According to the authors, this instrument improves discrimination between autism and other developmental problems. The M-CHAT reported sensitivity and specificity  .87, and  .99, respectively, positive predictive power of  .80 and negative predictive power  .99. [5] The sensitivity and specificity of the M-CHAT was determined by using 2 criteria: (1) failing 2 critical items (critical) or more of the 6 critical and (2) failing 3 or more critical items (if any) of the 23 total. According to the authors [5] the sensitivity and specificity for criterion 1 was  .97 and  .95, and criterion 2 was  .95 and  .99, respectively. Internal consistency was adequate for the full list of symptoms (*α* = .85) and for the 6 critical items (*α* = .83).

Mexico needs reliable and valid screening instruments for autism for use in the primary level of health care and education services. The purpose of this study was to investigate the psychometric properties of the Mexican (MM-CHAT-version) in a sample of referred young children with presumptive diagnosis of ASD and a sample from the general population.

## 2. Material and Methods

### 2.1. Participants

Children from 2 different settings participated in the study.

- Clinical sample. Cases with a presumptive diagnosis of ASD (Autism, Asperger's Disorder, PDD-NOS) were referred (*n* = 117) by the attending child psychiatrist of the PDD and ADHD outpatient clinic.
  1. An expert child psychiatrist conducted a semistructured clinical interview based on DSM-IV criteria for assigning the ASD diagnosis (Autism, Asperger PDD-NOS) and ADHD (subtypes inattentive, hyperactive-impulsive, and combined)) and the most common comorbidity (e.g., tics, Tourette disorder, generalized anxiety, phobia, oppositional disorder and conduct disorder, dysthymia).
  2. A senior board certified child psychiatrist with 20 years of experience administered the ADI-R.
- Children with typical development (*n* = 339) were recruited from nurseries located in four different districts of the city. Parents and teachers agreed to participate in the study after receiving a detailed description of the project. All parents from both samples completed the MM-CHAT and CBCL/1.5–5.

The inclusion criteria were children from both sexes between 18 to 72 months of age with a presumptive diagnosis of Autism Spectrum Disorder (Autism, Asperger's Disorder, PDD-NOS) and children from the general community with the same age range than the clinical group. Children were excluded if they had known comorbid severe chronic diseases that had the potential to bias the MM-CHAT scores, such as asthma, diabetes, cancer or sensory impairments such as deafness, blindness or a genetic syndrome associated with autism such as tuberous sclerosis, Rett syndrome, or fragile X.

### 2.2. Measures

#### 2.2.1. M-CHAT (Robins et al., 2001) [5]

The M-CHAT is a brief, simple instrument which takes about 15 minutes to complete. It consists of 23 items. The M-CHAT was developed by translating each item into Spanish and then adding minor cultural adjustments, such as describing the “peek-a-boo” game, since Mexican mothers do not have a specific name for it. For the purpose of this validation we used the following scores

1. The sum total of the items failed: MM-CHAT-T.
2. The total sum of the 6 critical items proposed in the literature = MM-CHAT-6ci criteria with the cutoff suggested by the authors.
  - Two or more critical items (2/6) failed = MM-CHAT-2/6.
  - Any three or more failed items (3/23) failed = MM-CHAT-3/23.

#### 2.2.2. Child Behavior Checklist, CBCL/1.5–5 (Achenbach and Rescorla, 2000) [22]

The CBCL/1.5–5 contains PDD, withdrawn and ADHD subscales. It consists of 100 emotional and behavioral problem items that are common in preschoolers. The results are grouped into the following syndromes: emotional reactivity, depression, anxiety, somatic complaints, attention problems, aggressive behavior, and sleep problems. In addition, the items are organized into three general scales of problems: total, externalized, and internalized. DSM also contains scales that assess the problems: mood, anxiety, developmental, attention-deficit hyperactivity, oppositional, and defiant behavior. The consistency of the scale is very high at  .95 and test-retest reliability is  .90.

The items are arranged in a Likert-type scale. Possible responses range from 0 = not applicable or never, 1 = sometimes, 2 = almost always.

In 2008 the scale was adapted and validated in Mexico [23]. For this study, we used the PDD, withdrawal and the ADHD subscales of the CBCL/1.5–5.

#### 2.2.3. Autism Diagnostic Interview (Autism Diagnostic Interview-Revised) (ADI-R) (Lord et al., 1994) [24]. 

The ADI-R is a semistructured interview that should be administrated by a clinician with experience evaluating children with autism. It is the gold standard for autism diagnosis of children and adults with mental ages older than 18 months [24]. The interview is organized according to the DSM-IV criteria. It contains 93 questions to explore the child's developmental history and questions that investigate problems associated with autism. The ADI-R algorithm generates scores for the three main domains of autistic symptomatology: (A) qualitative problems of reciprocal social behavior, (B) delayed language development, and (C) stereotyped behaviors and restrictive interests. It has an interrater reliability of  .83 to  .94.

In addition, the autism diagnosis in the clinical group was confirmed through a semistructured interview with DSM-IV criteria and the Autism Diagnostic Interview (ADI-R). Clinicians who conducted the interviews were blind to the questionnaire results. Inconsistency between both criteria was solved by consensus.

### 2.3. Ethical Issues

The study received approval from the hospital research committee. Written informed consent to participate in the study was obtained from each child's caregiver.

### 2.4. Statistical Analysis

#### 2.4.1. Demographics Variables Analysis

The demographic and clinical characteristics were expressed as means, standard deviations, and proportions. Student *t*-test was used to compare continuous variables such as children and parents age and socioeconomic status (SES).

#### 2.4.2. Reliability and Internal Consistency

Internal consistency was evaluated using the Kuder-Richardson coefficient for the total items (23) of MM-CHAT and the 6 critical items (2, 7, 9, 13, 14, 15) identified by the original validity study [5].

#### 2.4.3. Convergent Validity

Convergent validity was analyzed by calculating the Spearman correlations between the total score of the CBCL/1.5/PDD/withdrawn and ADHD subscales and the total score of the MM-CHAT failed items.

#### 2.4.4. Discriminant Validity

To investigate the discriminant validity we use *t*-test and chi square to analyse mean and percentage differences between the TD and ASD group ratings for the MM-CHAT-T and the percentage of items failure rate.

#### 2.4.5. Criterion Validity

The kappa coefficient (*κ*) was used to analyze the concordance between the categorical diagnosis of autism instruments obtained by ADI-R and the MM-CHAT using two cutoffs: (1) two or more critical items (2/6) failed (MM-CHAT-2/6) and (2) three or more any 23 critical items (3/23) failed (MM-CHAT-3/23).

## 3. Results

### 3.1. Demographic and Clinical Sample

Study participants were 456 children (74% male) with a range of 1–7 years and a mean age of 4.46 years (SD = 1.12). The sample was divided into two groups: (1) ASD (*n* = 117) and (2) typical development (TD) (*n* = 339). The groups were very similar for maternal age (ASD: *M* = 32.12, SD 6.80, compared to TD: *M* = 31.46, SD 7.10), paternal age (ASD: *M* = 36.51, SD 7.83, compared to TD: *M* = 36.06, SD 7.71), and socioeconomic status (ASD: *M* = 5.99 SD 2.92; against TD: *M* = 7.39, SD 6.64). However, the proportion of males was higher in the ASD group compared to the TD group (76.1% versus 51.91%). This difference was significant as shown in Table 1.

### 3.2. Internal Consistency

Internal consistency of the MM-CHAT for 23 items of the total sample was KR = .76 and for the 6 items (MM-CHAT-6ci) was KR = .70.

### 3.3. Convergent Validity

In the ASD group convergent validity was assessed using Spearman correlation coefficient (Rho) between the MM-CHAT-T, MM-CHAT-6ci and the CBCL/1.5–5/PDD, withdrawn and ADHD subscales and the ADI-R dimensions (*A*, *B*, and *C*). As shown in Table 2, correlations were varied. Dimension *B* (nonverbal) of the ADI-R had the highest correlation with the MM-CHAT-T (rho = 0.636, *P* ≤ .01) and the CBCL1.5–5/withdrawn subscale (rho = .66, *P* ≤ .01).

The MM-CHAT-6ci showed the highest correlation with the ADI-R A domain (rho = .66, *P* ≤ 0.01) and subscale CBCL1.5–5/withdrawn subscale as shown in Table 2. In the TD group Spearman correlations between the MM-CHAT and CBCL/1.5–5/PDD and withdrawn subscales were very low and nonsignificant (rho = .105, *P* < .19, rho = .073, *P* < .26) in the TD showed in Table 2.

### 3.4. Discriminant Validity

**Figure 1:**
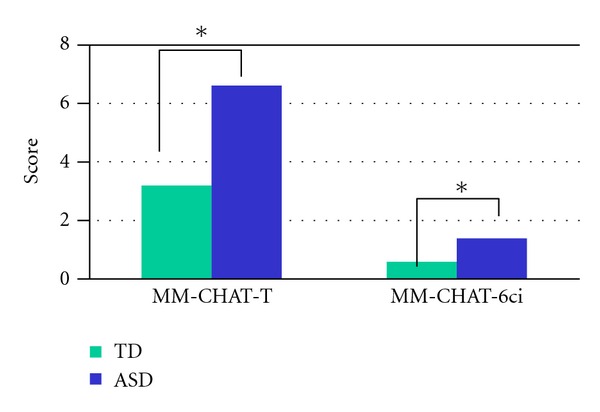
Groups differences for MM-CHAT.

The results are shown in Figure 1. The total score of the MM-CHAT was higher for the ASD group (*M* = 6.66, SD 4.21) compared to TD group (*M* = 3.27, SD 2.19), this difference was statistically significant (*P* ≤ .0001), see Table 3. By age group, the youngest group (1–3 years) had a higher mean score of the MM-CHAT (total failed items) for the ASD group (*M* = 5.44, SD 3.77) against TD (*M* = 2.41, SD 1.71, *P* ≤ .004), *F* = 20,904 (*t* = 3.30, df = 78, *P* ≤ .004). For the older group (4–6 years) the average score of the MM-CHAT (total failed items) for the groups was ASD (*M* = 5.97, SD 3.90) against TD (*M* = 3.44, SD 2.25), with a statistically significant difference (*F* = 49.90, *t* = 7.23, df = 343, *P* ≤ .0001). The average MM-CHAT-6ci for the groups was ASD (*M* = 1.44, SD 1.51) against TD (*M* 0.66, SD. 89), with a statistically significant difference between groups (*P* ≤ .0001).

**Figure 2:**
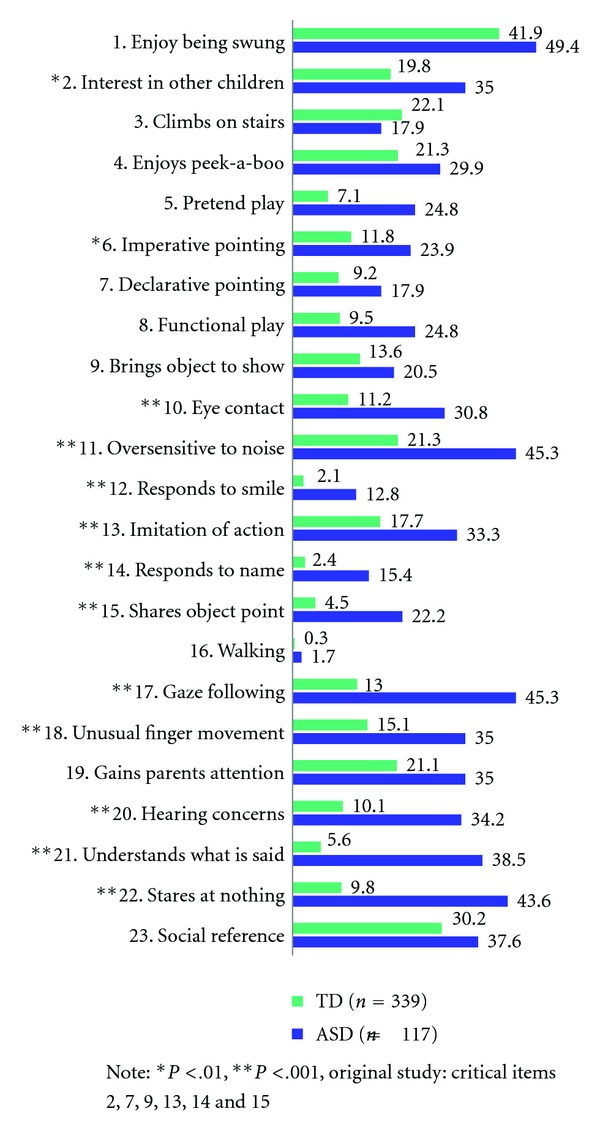
Failure rate for ASD and TD groups.

The chi-square test identified the significant failure rate for the ASD group and TD (see Figure 2) for the following 17 items (in bold): no. **2** (interest in other children) 35 versus 19.8, no. **6** (imperative pointing) 23.9 versus 11.8, no. **10** (eye contact) 30.8 versus 11.2, no. **11** (noise) 45.3 versus 21.3, no. **12** (responds to smile) 12.8 versus 2.1, no. **13** (imitation) 33.3 versus 17.7, no. **14**. (response to name) 15.4 versus 2.4, no. **15** (shares object point) 22.2 versus 4.5, no. **16** (walk) 1.7 versus 0.3, no. **17** (gaze following) 45.3 versus 13, no. **18** (unusual finger movements) 35 versus 21.1, no. **20** (hearing concerns) 34.2 versus 10.1, no. **21** (understands what is said) 38.5 versus 5.6, no. **22** (stares at nothing) 43.6 versus 9.8.

### 3.5. Construct Validity

To analyze the construct validity of the MM-CHAT Mexican version, we calculated the kappa coefficient (*κ*) in the ASD group using the following criteria: 

MM-CHAT 2/6 or greater (cutoff suggested in the original study; Robins, et al., 2001) [5]. The criterion for detection of autism is failing two or more of the 23 items (2 or more)

- ADI-R (gold standard) dichotomic scoring of dimensions *A*, *B*, and *C*.
- ADI-R (gold standard) categorical diagnosis of autism. In the ASD group kappa coefficient (*κ*) between the MM-CHAT and the ADI-R dimensions was (*κ*) =  .17 to  .61 as shown in Table 3.

## 4. Discussion

This study investigated the psychometric properties of the MM-CHAT Spanish version for Mexico in two different samples: clinical and TD from the general community. Most validation studies of the M-CHAT [25, 26] including the original [5] have used large samples of the general population to identify a very small number of children with autism (*n* = 4 and *n* = 7, resp.). In this study we used a case control design which included a large clinical group of children who were seen in the outpatient PDD clinic before an autism diagnosis was assigned. Overall the MM-CHAT could discriminate between the TD and the ASD group. The instrument showed moderate internal consistency and convergent validity with CBCL/1.5–5. However, there are some results which deserve a more detailed analysis. ADI-R Spearman correlations with MM-CHAT scores were varied (rho = .23–.66), this result is consistent with the notion that autism is a complex and heterogeneous disorder. However, overall MM-CHAT-T correlations were better than the MM-CHAT6ci particularly for the ADI-R (BV) and *C* dimensions (rho  .23, *P* < .07 and rho  .36, *P* < .01), this gain represents a small drop in the *A* dimension correlation from  .66 to  .61 with the same *P* < .01 value. An addition of items exploring communication abnormalities such as echolalia, language loss and/or delay salient aspects of autism could make the BV correlation rise and become significant.

All kappas were significant using the MM-CHAT-2 criteria (best cutoff in this study) and the ADI-R cutoff domains except for the dimension *C*(*κ*).17 *P* = .09.   The MM-CHAT-T had a higher concordance with the ADI-R nonverbal dimension (BNV) (*κ*)  .61 *P* = .0001 in contrast to the *B* verbal (BV) dimension and the *C* dimension of stereotypical behavior (*κ*).29 *P* = .004, and (*κ*) .16 *P* = .01. This result supports the idea suggested by other researchers that the M-CHAT detects better nonverbal children with low functioning autism [27, 28]. 

The MM-CHAT showed discriminant validity between the ASD and the TD group through analyzing differences on the MM-CHAT-T means and percentage of failed items. Critical items in this study are not the same as the one proposed in the original study.

The detection of the Critical items has been inconsistent in the studies [5, 26]. Some factors such as the sample composition (clinical versus community) or age range and the statistical procedures to derive them could explain these differences. Nevertheless, there is growing evidence from studies with combined samples from different race composition which suggests a cultural bias does exist for autistic measures like M-CHAT [29] and other standardized measures for autism [30]. 

However, as more international studies of validation are published it is becoming evident that the M-CHAT has important differences in items that parents endorsed more frequently. The reason for this cultural bias is unclear, but it is possible that differences in parenting and social behavior styles could be influencing this phenomenon. Many rating scales for autism are dichotomic because they were developed when autism was understood as a categorical disorder. Recent evidence suggests that autism traits are normally distributed in the general population [30–36] and that not only parents, but also individuals without autism in their families express these traits. Based on this latter evidence, the suggestion made by some researchers [26, 37] to modify the M-CHAT as a quantitative measure with items reorganized as a likert scale type seems appropriate. In 2007, a supplementary telephone interview for parents of children who screened positive was developed [38]. The combined use of the M-CHAT screen and the telephone interview increases its positive predictive value without adversely affecting its sensitivity [38]. However, this incremental validity will raise the cost of M-CHAT excessively for countries like Mexico. There are important reasons to screen and take action at the same time. The addition of an interview requires training which can be very difficult to give and maintain in busy settings.

As in other studies, we also observed that some parents do not understand all of the MM-CHAT questions [25]. This has been attributed to a low education level of the caregiver. There is incipient evidence that contradicts this idea [39]. Autism signs are bizarre, elusive and some of them transient, so even highly educated parents can miss these symptoms. Parents might believe it to be irrelevant if their children do not point to share pleasure. Furthermore, parents of children with autism often share some autistic traits and there is evidence of assortative mating for these traits among spouses [40, 41]. It is unknown if having these traits can weaken the parents' abilities to detect them in their children. In some Asian and Latin-American cultures like Mexico, making eye contact is considered inappropriate and a sign of disrespect. Some mothers discourage children from pointing with the index finger because it is considered to be rude [42, 43]. 

These cultural issues could explain some of the inconsistency in items responses independent of the instrument [44]. For these reasons, developmental assessment is one of the most challenging tasks for service providers and parents. The addition of pictograms employed by Inada et al., 2011 [26] is a proposal that deserves more investigation. Up to date no prevalence studies on autism have been done in Mexico. In this study, 5% of parents in the TD group met the MM-CHAT-2 criteria. This figure gives support to the urgent need to develop and/or validate gold standard instruments to confirm an autism diagnosis.

## 5. Conclusions

This study has some limitations. Despite having a large sample of undiagnosed ASD children from the psychiatric outpatient PDD clinic, we were unable to include the IQ tests results and analyze the effect of this variable on the MM-CHAT cutoff performance. The majority of young children were unmedicated, but others with challenging behaviors had medication for their hyperactivity and irritability, which could bias some of the parents' responses. Overall, in this study we demonstrated that the MM-CHAT can discriminate between ASD and TD children. The instrument has good psychometric properties and can be used for screening purposes in primary settings or busy specialized psychiatric clinics. However, these results support evidence for cultural differences in item responses, making it difficult to compare M-CHAT results internationally. 

## Figures and Tables

**Table 1:** Groups demographics.

Variables	TD	ASD	*P*
	*n* = 339	*n* = 117	
Sex* n *(%)			
Male	176 (51.9)	89 (76.1)	.0001
Age* M *(SD)			
Children	4.48 (1.13)	4.40 (1.11)	NS
Mother	31.46 (7.10)	32.12 (6.80)	NS
Father	35.06 (7.71)	36.51 (7.83)	NS
SES* M *(SD)	7.39 (6.64)	5.99 (2.92)	NS

Note. TD: typical development, ASD: autism spectrum disorder, *M*: mean, SD: standard deviation, NS: nonsignificant, SES: socioeconomic status.

**Table 2:** Convergent validity. Spearman correlations between MM-CHAT, ADI-R, and CBCL/1.5–5 of ASD group.

Variables		**1**	**2**	**3**	**4**	**5**	**6**	**7**	**8 **
MM-CHAT	(1) *T*	1							
	(2) 6ci	.85	1						

ADI-R	(3) *A*	.61**	.66**	1					
	(4) BV	.23	.08	.42**	1				
	(5) BNV	.63**	.61**	.83**	.58**	1			
	(6) *C*	.36**	.25*	.40**	.49**	.49**	1		

CBCL/1.5–5	(7) PDD	.65**	.56**	.56**	.18	.49**	.34**	1	
	(8) *W*	.66**	.63**	.60**	.24	.55**	.20	.86**	1

Note. **P ≤ .01, *P < .05*. T*: MM-CHAT total recoded failed items, score, 6CI: sum of MM-CHAT six critical items, *A*: total score of ADI-R A1 + A2 + A3 + A4 items, BV: total score of ADI-R B1 + B4 + B2 (verbal) + B3 (verbal) del ADI-R. BNV: total score of ADI-R B1 + B4, *C*: total score of ADI-R C1 + C2 + C3, *W* = CBCL/1.5–5/withdrawn, PDD = CBCL/1.5–5/pervasive developmental scale.

**Table 3:** Kappa (*κ*) coefficient of MM-CHAT and ADI-R in ASD group.

MM-CHAT dichotomic score using any two critical ítems		
ADI-R (cutoff point)	Kappa (*κ*)	*P*
*A* (10)	.168	**.019**
*B* verbal (8)	.290	**.004**
*B* nonverbal** (7)**	**.614**	**.0001**
*C* (3)	.175	.095

## References

[1] Baird G, Simonoff E, Pickles A (2006). Prevalence of disorders of the autism spectrum in a population cohort of children in South Thames: the Special Needs and Autism Project (SNAP). *Lancet*.

[2] Baron-Cohen S, Scott FJ, Allison C (2009). Prevalence of autism-spectrum conditions: UK school-based population study. *British Journal of Psychiatry*.

[3] Kawamura Y, Takahashi O, Ishii T (2008). Reevaluating the incidence of pervasive developmental disorders: impact of elevated rates of detection through implementation of an integrated system of screening in Toyota, Japan. *Psychiatry and Clinical Neurosciences*.

[4] Rice C, Nicholas J, Baio J (2010). Changes in autism spectrum disorder prevalence in 4 areas of the United States. *Disability and Health Journal*.

[5] Robins DL, Fein D, Barton ML, Green JA (2001). The Modified Checklist for Autism in Toddlers: an initial study investigating the early detection of autism and pervasive developmental disorders. *Journal of Autism and Developmental Disorders*.

[6] Osterling J, Dawson G (1994). Early recognition of children with autism: a study of first birthday home videotapes. *Journal of Autism and Developmental Disorders*.

[7] Hurlh J, Shaw E, Izeman SG, Whaley K, Rogers SJ (1999). Areas of agreement about effective practices among programs serving young children with autism spectrum disorders. *Infants and Young Children*.

[8] Smith T (1999). Outcome of early intervention for children with autism. *Clinical Psychology: Science and Practice*.

[9] Harris SL, Delmolino L (2002). Applied behavior analysis: its application in the treatment of autism and related disorders in young children. *Infants and Young Children*.

[10] Matson JL (2007). Current status of differential diagnosis for children with autism spectrum disorders. *Research in Developmental Disabilities*.

[11] Matson JL (2007). Determining treatment outcome in early intervention programs for autism spectrum disorders: a critical analysis of measurement issues in learning based interventions. *Research in Developmental Disabilities*.

[12] Sutera S, Pandey J, Esser EL (2007). Predictors of optimal outcome in toddlers diagnosed with autism spectrum disorders. *Journal of Autism and Developmental Disorders*.

[13] Dawson G, Rogers S, Munson J (2010). Randomized, controlled trial of an intervention for toddlers with autism: The early start Denver model. *Pediatrics*.

[14] Bishop D How to Choose a diagnostic tools? Discussion: a plea for efficiency. http://psyweb.psy.ox.ac.uk/oscci/.

[15] Matson JL, Rieske RD, Tureck K (2011). Additional considerations for the early detection and diagnosis of autism: review of available instruments. *Research in Autism Spectrum Disorders*.

[16] Akshoomoff N, Corsello C, Schmidt H (2006). The role of the autism diagnostic observation schedule in the assessment of autism spectrum disorders in school and community settings. *The California School Psychologist*.

[17] Volkmar FR, Stier DM, Cohen DJ (1985). Age of recognition of pervasive developmental disorder. *American Journal of Psychiatry*.

[18] Kishore MT, Basu A (2011). Early concerns of mothers of children later diagnosed with autism: implications for early identification. *Research in Autism Spectrum Disorders*.

[19] de Autismo España e Instituto de Investigación de Enfermedades Raras (IIER) Confederación Informe sobre demora en el diagnostico en los TEA. http://iier.isciii.es/autismo/pdf/aut_isdd2.pdf.

[20] Talero-Gutiérrez C, Rodríguez M, De La Rosa D, Morales G, Vélez-Van-Meerbeke A (2011). Profile of children and adolescents with autism spectrum disorders in an institution in Bogota, Colombia. *Neurologia*.

[21] Yeargin-Allsopp M, Rice C, Karapurkar T, Doernberg N, Boyle C, Murphy C (2003). Prevalence of autism in a US metropolitan area. *Journal of the American Medical Association*.

[22] Achenbach TM, Rescorla LA Manual for the ASEBA Preschool Forms & Profiles.

[23] Albores-Gallo L, Hernández-Guzmán  L, Díaz-Pichardo J, Cortes-Hernández B, Esperón-Vargas C Validity and reliability of CBCL/1.5-5 Mexican version.

[24] Lord C, Rutter M,  Le Couteur A (1994). Autism Diagnostic Interview-Revised: a revised version of a diagnostic interview for caregivers of individuals with possible pervasive developmental disorders. *The Journal of the American Medical Association*.

[25] Perera H, Wijewardena K, Aluthwelage R (2009). Screening of 18-24-month-old children for autism in a semi-urban community in Sri Lanka. *Journal of Tropical Pediatrics*.

[26] Inada N, Koyama T, Inokuchi E, Kuroda M, Kamio Y (2011). Reliability and validity of the Japanese version of the Modified Checklist for autism in toddlers (M-CHAT). *Research in Autism Spectrum Disorders*.

[27] Buitelaar J, Beuker K, Schjolberg S, KveimLie K, Hornig M, Bresnahan M M-CHAT and ESAT screening questionnaires at 18 months in the general population: issues of overlap and external validity.

[28] VandenBerg A, Lecavalier L A comparison of the modified checklist for autism in toddlers and the social communication questionnaire in preschoolers suspected of having pervasive developmental disorders.

[29] Reyes NM, Patriquin MA, Scarpa A, Kerkering K Differences between English- and Spanish-Speaking mother's report on Toddler's profiles in the modified checklist for Autism in Toddlers (M-CHAT).

[30] Joseph L, Shumway S, Thurm A Repetitive behaviors in young children with autism: specificity and stability.

[31] Baron-Cohen S (1995). *Mindblindness: An Essay on Autism and Theory of Mind*.

[32] Piven J, Palmer P, Jacobi D, Childress D, Arndt S (1997). Broader autism phenotype: evidence from a family history study of multiple-incidence autism families. *American Journal of Psychiatry*.

[33] Spiker D, Lotspeich LJ, Dimiceli S, Myers RM, Risch N (2002). Behavioral phenotypic variation in autism multiplex families: evidence for a continuous severity gradient. *American Journal of Medical Genetics*.

[34] Constantino JN, Todd RD (2003). Autistic traits in the general population: a twin study. *Archives of General Psychiatry*.

[35] Hoekstra RA, Bartels M, Verweij CJH, Boomsma DI (2007). Heritability of autistic traits in the general population. *Archives of Pediatrics and Adolescent Medicine*.

[36] Skuse DH, Mandy W, Steer C (2009). Social communication competence and functional adaptation in a general population of children: preliminary evidence for sex-by-verbal IQ differential risk. *Journal of the American Academy of Child and Adolescent Psychiatry*.

[37] Wong V, Hui L, Lee W (2004). A modified screening tool for autism (Checklist for Autism in Toddlers [CHAT-23]) for Chinese children. *Pediatrcs*.

[38] Kleinman JM, Robins DL, Ventola PE (2008). The modified checklist for autism in toddlers: a follow-up study investigating the early detection of autism spectrum disorders. *Journal of Autism and Developmental Disorders*.

[39] Glascoe FP, Macias MM, Wegner LM, Robertshaw NS (2007). Can a broadband developmental-behavioral screening test identify children likely to have autism spectrum disorder?. *Clinical Pediatrics*.

[40] Virkud YV, Todd RD, Abbacchi AM, Zhang Y, Constantino JN (2009). Familial aggregation of quantitative autistic traits in multiplex versus simplex autism. *American Journal of Medical Genetics B*.

[41] Constantino JN, Todd RD (2005). Intergenerational transmission of subthreshold autistic traits in the general population. *Biological Psychiatry*.

[42] Non-Verbal Communication Modes. http://www2.andrews.edu/~tidwell/bsad560/NonVerbal.html.

[43] Morris D Bodytalk: The Meaning of Human Gestures. New York: Crown Trade Paperbacks. Non-verbal Communication. Providers Guide to Quality and Culture. http://erc.msh.org/mainpage.cfm?file=4.6.0.htm&module=provider&language=English.

[44] Pinto-Martin JA, Young LM, Mandell DS, Poghosyan L, Giarelli E, Levy SE (2008). Screening strategies for autism spectrum disorders in pediatric primary care. *Journal of Developmental and Behavioral Pediatrics*.

